# Supplementary material for: A paradoxical relationship between mitochondrial calcium regulation and retinal ganglion cell degeneration after axon damage
Source: bioRxiv. 2026 May 15:2026.05.13.724793. Preprint. [Version 1] doi: 10.64898/2026.05.13.724793 (PMC13193050; doi:10.64898/2026.05.13.724793)
Supplement: Supplement 1 [file NIHPP2026.05.13.724793v1-supplement-1.pdf]

Supplemental Figure Captions

Supplemental Figure 1. Ru265 decreases Mito-Ca^{2+} levels in a dose dependent manner. (A) Example *in vivo* 2-photon aips of mito-T2b before and 10 min after injection of vehicle control (50% DMSO and 50% PBS). (B) Line graphs of mito-Ca^{2+} levels before and 10 min after vehicle injection. Individual RGCs are in grey, and mean is in black, MWU test (n = 95 RGCs from 3 retinas and 3 mice). (C) Swarm plot of changes in mito-Ca^{2+} levels after vehicle injection. (D) Example *in vivo* 2-photon aips of mito-T2b before and 10 min after injection of 20 μM Ru265. Arrow indicates RGC with reduction in mito-Ca^{2+}. (E) Line graphs of mito-Ca^{2+} levels before and 10 min after 20 μM Ru265 injection. Individual RGCs are in grey, and mean is in black, MWU test (n = 101 RGCs from 4 retinas and 4 mice). (F) Swarm plot of changes in mito-Ca^{2+} levels after 20 μM Ru265injection. (G) Example *in vivo* 2-photon aips of mito-T2b before and 10 min after injection of 200 μM Ru265. Arrows indicate RGCs with reduction in mito-Ca^{2+}. (H) Line graphs of mito-Ca^{2+} levels before and 10 min after 200 μM Ru265 injection. Individual RGCs are in grey, and mean is in black, MWU test (n = 159 RGCs from 4 retinas and 4 mice). (I) Swarm plot of changes in mito-Ca^{2+} levels after 200 μM Ru265 injection. Scale bars = 50 μm.

Supplemental Figure 2. Ru265 induces Ca^{2+} elevations at 2 minutes post injection, likely due to DMSO vehicle. (A) Example *in vivo* 2-photon aips of mito-T2b before and 2 min after injection of 2 mM Ru265 (left). Line graphs of mito-Ca^{2+} levels before and 2 min after 2 mM Ru265 injection. Individual RGCs are in blue, and mean is in black (n = 154 RGCs from 4 retinas and 4 mice). (B) Example *in vivo* 2-photon aips of cyto-T2b before and 2 min after injection of 2 mM Ru265 (left). Line graphs of cyto-Ca^{2+} levels before and 2 min after 2 mM

bioRxiv preprint doi: https://doi.org/10.64898/2026.05.13.724793; this version posted May 15, 2026. The copyright holder for this preprint (which was not certified by peer review) is the author/funder, who has granted bioRxiv a license to display the preprint in perpetuity. It is made available under a CC-BY 4.0 International license.

Ru265 injection. Individual RGCs are in orange, and mean is in black (n = 96 RGCs from 4 retinas and 4 mice). (C) Swarm plots comparing changes in Ca^{2+} levels 2 min after 2 mM Ru265 between cyto- and mito-T2b. Black bars are mean +/- SEM, MWU test. (D) Example *in vivo* 2-photon aips of mito-T2b before and 2 min after injection of DMSO/PBS vehicle (left). Line graphs of mito-Ca^{2+} levels before and 2 min after vehicle injection. Individual RGCs are in blue, and mean is in black (n = 73 RGCs from 3 retinas and 3 mice). (E) Example *in vivo* 2-photon aips of cyto-T2b before and 2 min after injection of behicle (left). Line graphs of cyto-Ca^{2+} levels before and 2 min after vehicle injection. Individual RGCs are in orange, and mean is in black (n = 87 RGCs from 3 retinas and 3 mice). (F) Swarm plots comparing changes in Ca^{2+} levels 2 min after vehicle injection between cyto- and mito-T2b, MWU test. Scale bars = 50 μm.

Supplemental Figure 3. Mitochondrial targeted Twitch2b mislocalization occurs after longer expression durations. (A) Example confocal mips of fixed retinal wholemount showing endogenous mito-T2b expression (green) and TOMM20 immunostaining (magenta) at the indicated time points after AAV injection. Yellow arrow indicates RGC with partial mislocaliztion and orange arrows indicate RGCs with complete mislocaliztion of mito-T2b. (B) Example confocal mips of ‘healthy’ RGCs expressing mito-T2b co-localized with TOMM20, co-stained with RBPMS (blue). (C) Example confocal mips of RGCs expressing mito-T2b that is ‘partially mislocalizated’ and immunostained for TOMM20 and RBPMS. (D) Example confocal mips of RGCs expressing mito-T2b that is ‘completely mislocalizated’ and immunostained for TOMM20 and RBPMS. (E-F) Pie charts showing the proportion of RGCs in each of the qualitative mito-T2b localization groups at indicated time points after AAV injection (< 30 dpi n = 917 RGCs from 5 retinas and 3 mice; > 60 dpi n = 446 RGCs from 4 retinas from 4 mice mice). (G) Percentage of RGCs in indicated mislocalization groups at indicated time points. Graph is sample means +/- SEM (MWU test). (H-I) Z-score of mito-T2b expression intensity measure from fixed wholemounts representing indicated qualitative localization groups and time points after AAV injection. Graph is sample means +/- SEM. Scale bars = 10 μm.

bioRxiv preprint doi: https://doi.org/10.64898/2026.05.13.724793; this version posted May 15, 2026. The copyright holder for this preprint (which was not certified by peer review) is the author/funder, who has granted bioRxiv a license to display the preprint in perpetuity. It is made available under a CC-BY 4.0 International license.

Supplemental Figure 4. Endoplasmic Reticulum Ca^{2+} levels are not correlated with survival to ONC. (A) Example *in vivo* 2-photon mips of cytoplasmic cyto-T2b (left) and ER-2b (right) expressed in RGCs of VGlut2-Cre mice. (B) Swarm plots of homeostatic cyto-, mito-, and ER-Ca^{2+} levels, as measured by cpVenus (YFP) to mCerulean (CFP) FRET ratios (cyto-T2b and mito-T2b replotted from Figure 1; ER-T2b = 717 RGCs from 9 retinas and 5 mice. MWU test). (C) Example *in vivo* 2-photon aips of ER-Ca^{2+} expressing RGCs before ONC and at the indicated days post ONC (dpc). (D) Swarm plots of pre-injury ER-Ca^{2+} levels in RGCs that survived (green) or died (red) following ONC. Means +/- SEM are labeled with black bars (survive n = 28 RGCs from 4 retinas and 3 mice, die n = 133 RGCs. MWU test). Scale bars = 100 μm.

Supplemental Figure 5. Chronic Ru265 treatment does not kill RGCs. (A) Representative mips of retinal wholemounts immunostained with RBPMS at 14 days after sham ONC and indicated treatments. (B) Quantification of RBPMS densities from indicated experimental conditions. Points are individual retinas bars are mean +/- SEM (Sham ONC + Vehicle n = 5 retinas from 4 mice; Sham ONC + Sham injection n = 4 retinas from 4 mice; Sham ONC + Ru265 n = 5 retinas from 4 mice). (C) Representative mips of retinal wholemounts immunostained with RBPMS at 14 days after ONC and indicated sham treatments. (D) Quantification of RBPMS densities from indicated experimental conditions. Points are individual retinas bars are mean +/- SEM (ONC + Sham n = 6 retinas from 6 mice; ONC + PBS n = 5 retinas from 5 mice; ONC + Vehicle n = 6 retinas from 6 mice). Scale bars = 100 μm.

Supplemental Figure 6. Validation of AAV mediated manipulation of MCU expression. (A) Example confocal mips of retinal wholemounts immunostained for MCU (green) and RBPMS (blue) with endogenous mCherry reporter signal from AAV-MCU-OX treatment (red). (B) Scatterplot of MCU immunofluorescence intensity and mCherry reporter signal measured from confocal wholemount images. Dashed line represents trendline for all RGCs with a 95%

bioRxiv preprint doi: https://doi.org/10.64898/2026.05.13.724793; this version posted May 15, 2026. The copyright holder for this preprint (which was not certified by peer review) is the author/funder, who has granted bioRxiv a license to display the preprint in perpetuity. It is made available under a CC-BY 4.0 International license.

confidence interval. Grey background shading indicates threshold for flagging RGCs as mCherry- or mCherry+. (C) Box and whisker plaots of MCU immunostaining intensity compared between MCU-OX mCherry reporter positive and negative RGCs. Points are individual RGCs, boxes depict the lower quartiles, the median, and the upper quartiles while the whiskers represent the minimum and maximum datapoint at 1.5 x the interquartile range. MWU test (n = 504 RGCs from 3 retinas and 3 mice). (D) Example confocal mips of retinal wholemounts immunostained for MCU (green) and RBPMS (blue) with endogenous mCherry reporter signal from AAV-shMCU treatment (red). (E) Scatterplot of MCU immunofluorescence intensity and mCherry reporter signal measured from confocal wholemount images. Dashed line represents trendline for all RGCs with a 95% confidence interval. Grey shading indicates threshold for flagging cells as mCherry- or mCherry+. (F) Box and whisker plots of MCU immunostaining intensity compared between shMCU mCherry reporter positive and negative RGCs. Points are individual cells, boxes depict the lower quartiles, the median, and the upper quartiles while whiskers represent the minimum and maximum datapoint at 1.5 x the interquartile range, MWU test (n = 489 RGCs from 3 retinas and 3 mice). Scale bars = 100 μm.

Supplemental Figure 7. MCU-OX leads to slow RGC degeneration. (A) Representative mips of fixed retinal wholemounts immunostained for RBPMS at 14 days after sham surgery. (B) Quantification of RBPMS+ cell density in mice with intravitreal injections of indicated AAV MCU gene expression manipulations compared to littermate AAV-mCherry treated controls. Points indicate individual eyes, bars indicate mean +/- SEM. (mCherry n = 6 retinas from 6 mice, shMCU n = 3 retinas from 3 mice, MCU-OX n = 7 retinas from 7 mice). (C) Representative mips of fixed retinal wholemounts immunostained for RBPMS at 8 weeks after sham surgery. (D) Quantification of RBPMS+ cell density in mice with intravitreal injections of indicated AAV MCU gene expression manipulations compared to littermate AAV-mCherry treated controls. Points indicate individual eyes, bars indicate mean +/- SEM, MWU test. (mCherry n = 4 retinas from 4

bioRxiv preprint doi: https://doi.org/10.64898/2026.05.13.724793; this version posted May 15, 2026. The copyright holder for this preprint (which was not certified by peer review) is the author/funder, who has granted bioRxiv a license to display the preprint in perpetuity. It is made available under a CC-BY 4.0 International license.

mice, shMCU n = 3 retinas from 3 mice, MCU-OX n = 3 retinas from 3 mice). Scale bars = 100 μm.
